# Supplementary material for: Causal Relationship between Aging and Anorexia Nervosa: A White-Matter-Microstructure-Mediated Mendelian Randomization Analysis
Source: Biomedicines. 2024 Aug 16;12(8):1874. doi: 10.3390/biomedicines12081874 (PMC11351342; doi:10.3390/biomedicines12081874)
Supplement: Supplementary file 1 [file biomedicines-12-01874-s001.zip › Supplementary Figure/Figure S8/Leaveoneout plot of MR analysis for TL to ubm-b-1902.pdf]

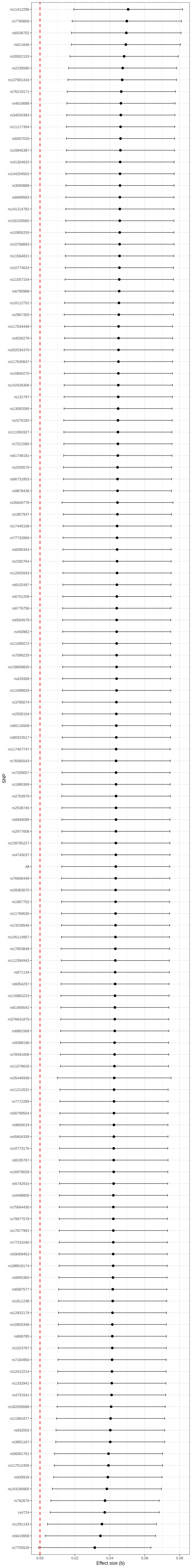

rs11412296
rs7790856
rs6536702
rs611646
rs28502153
rs2230590
rs137901416
rs76219171
rs4616688
rs34550383
rs11117354
rs6007020
rs10845387
rs41304832
rs144204502
rs3093888
rs6669563
rs141214782
rs150150565
rs10905255
rs10768683
rs11584821
rs10774624
rs11557154
rs6790988
rs10112752
rs2967355
rs117034449
rs4530278
rs202034370
rs117630647
rs10840270
rs142426306
rs131797
rs13062095
rs2276182
rs111950327
rs7221585
rs61748181
rs2293579
rs66731853
rs9878436
rs35640778
rs1957937
rs17445108
rs77732866
rs6590343
rs2282764
rs12925933
rs8102497
rs6751209
rs6776756
rs6584579
rs450962
rs11085072
rs7099229
rs139669835
rs429358
rs11699829
rs3785074
rs2555104
rs80116508
rs80324517
rs117407747
rs76065543
rs7209057
rs1985369
rs2763979
rs2538745
rs9940099
rs2977608
rs139795227
rs4743037
All
rs76666449
rs28363070
rs1907702
rs11769630
rs13230646
rs145114957
rs17803849
rs112394943
rs871134
rs6054257
rs116863223
rs61405042
rs376641875
rs6881568
rs9398196
rs78491606
rs11579626
rs35446936
rs11212631
rs7772289
rs56799554
rs9600019
rs45604339
rs10773176
rs8105767
rs16978028
rs5742915
rs4498805
rs75664430
rs79977579
rs17677991
rs77231040
rs59409453
rs188918174
rs9955360
rs6587577
rs1611236
rs12932179
rs10805346
rs869785
rs1023767
rs7164950
rs12412214
rs1332941
rs4731541
rs182059586
rs11991877
rs932002
rs3891167
rs56061761
rs117512405
rs939916
rs143190905
rs762679
rs4724
rs1291143
rs9419958
rs7705526
SNP
0.00
0.02
0.04
0.06
0.08
Effect size (b)
